# From API to Action: A Multi-Model Comparison of OpenAI, Anthropic, Google, and Meta LLMs for Clinical Trial Data Extraction

**DOI:** 10.3390/bioengineering13070773

**Published:** 2026-07-02

**Authors:** Richard J. Young, Jorge Fonseca, Brach Poston

**Affiliations:** 1 Interdisciplinary Neuroscience, University of Nevada, Las Vegas, NV 89154, USA; brach.poston@unlv.edu; 2 Department of Computer Science, Howard R. Hughes College of Engineering, University of Nevada, Las Vegas, NV 89154, USA; jorge.fonsecacacho@unlv.edu; 3 Department of Kinesiology and Nutrition Sciences, University of Nevada, Las Vegas, NV 89154, USA

**Keywords:** clinical trial data extraction, large language models, neuromodulation, transcranial direct current stimulation, API integration, systematic review methodology, Parkinson’s disease, evidence synthesis, research automation

## Abstract

**(1) Background:** Clinical trial data extraction from registries such as ClinicalTrials.gov remains labor-intensive and error-prone, often missing critical details hidden in unstructured protocol descriptions. Large Language Models (LLMs) offer potential to automate this process, yet systematic multi-model comparisons on real clinical trial data remain scarce. **(2) Methods:** Four LLMs (OpenAI o4-mini-high, Anthropic Claude-Sonnet-4, Google Gemini 2.5-Pro, and Meta Llama-4-Maverick) extracted brain stimulation parameters from 67 transcranial direct current stimulation (tDCS) trials in Parkinson’s disease via a structured JSON schema. Pairwise inter-model agreement was quantified with Cohen’s Kappa and percentage agreement across binary, categorical, and multi-component task tiers. **(3) Results:** Under exact-string matching, agreement was near-perfect for binary classifications (non-invasive classification: 100%; brain stimulation presence: 99.3%, κ = 0.50) and substantial for categorical extractions (primary stimulation type: 96.4%, κ = 0.70), but fell to 48.6% (κ = 0.43) for complex anatomical targets. Numeric parameters revealed model-specific strengths: o4-mini-high and Claude-Sonnet-4 achieved perfect duration agreement (*r* = 1.000, *n* = 19) while Llama-4-Maverick diverged substantially (*r* < 0.12). Validation against an expert gold standard (100% inter-annotator agreement on a 20-trial overlap) confirmed high extraction accuracy across all features (mean 93.7–98.9%). Crucially, the low agreement on anatomical targets proved to be an artifact of exact-string scoring: under the same semantic matching used to measure accuracy, inter-model agreement rose to 97.0%, coinciding with the 95.5% expert accuracy. Inter-model agreement therefore tracks accuracy once both are measured on a common basis. **(4) Conclusions:** Exact-string inter-model agreement decreases with task complexity, but this decline largely reflects interchangeable free-text wording rather than reduced accuracy. Evaluated semantically, agreement and expert accuracy are both high and closely aligned. A residual risk is not low accuracy but the rare error shared across all models, which agreement cannot detect, and which overall accuracy can itself mask when one class dominates. These findings inform hybrid human–AI systematic review pipelines in which targeted expert oversight focuses on shared-error and minority-class detection.

## 1. Introduction

Clinical trials constitute the foundation of evidence-based medicine, providing essential data on therapeutic safety and efficacy [1,2]. ClinicalTrials.gov, maintained by the National Library of Medicine at the National Institutes of Health, contains over 400,000 records from studies conducted worldwide, representing an invaluable repository of clinical evidence [3]. However, extracting and synthesizing data from these records remains a significant challenge. Traditional systematic reviews rely on keyword-based searches and manual data extraction, processes that are labor-intensive, prone to human error, and can require months to complete [4,5]. Critical information (such as stimulation parameters, dosing protocols, or patient stratification criteria) often resides in unstructured text fields, necessitating extensive human curation [6,7]. This bottleneck delays the translation of research findings into clinical practice and impedes timely updates to treatment guidelines. The growing volume and complexity of clinical trial data demand automated solutions capable of accurate, scalable extraction.

Large Language Models (LLMs) have emerged as promising tools for automating clinical data extraction [8]. Recent foundational models, including OpenAI’s o4-mini-high [9], Anthropic’s Claude-Sonnet-4 [10], Google’s Gemini 2.5-Pro [11], and Meta’s Llama-4-Maverick [12], exhibit capabilities extending well beyond conventional lexical matching. According to the New England Journal of Medicine AI [13], these models could transform scientific research. However, concerns regarding accuracy, reliability, and hallucinations persist [14,15].

Prior research has demonstrated LLM potential in clinical contexts [16,17]. Stuhlmiller et al. [18] developed a scalable method for validated data extraction from electronic health records, while Konet et al. [19] reported that Claude 2 and GPT-4 achieved high accuracy extracting pre-specified data elements from clinical trials. Ntinopoulos et al. [20] reported greater than 98% accuracy for 18 LLMs evaluated on synthetic cardiac notes, and Khan et al. [21] achieved 94% accuracy using collaborative LLM workflows on structured clinical trial variables. Most directly relevant, our group previously benchmarked five prior-generation LLMs for tDCS protocol extraction from aging-related trials [5]. The present study advances that line in three respects: it evaluates four current-generation frontier models (o4-mini-high, Claude-Sonnet-4, Gemini 2.5-Pro, and Llama-4-Maverick), targets Parkinson’s disease (PD) rather than aging cohorts, and, most importantly, introduces an explicit, complexity-stratified characterization of how inter-model agreement degrades across binary, categorical, and multi-component extraction tasks, a relationship not previously quantified.

This work sits within a rapidly expanding literature on LLM-based clinical evidence extraction, yet none of the recent studies occupies its specific intersection. A growing body of work pairs inter-model concordance with a human gold standard. Lai et al. [22] reported almost-perfect inter-model agreement (κ = 0.88) alongside 95–97% accuracy against a methodologist standard for complementary-medicine trials, and Liu et al. [23] coupled a structured multi-item schema with a 94.8% correctness rate. Both Courvoisier et al. [24] and van der Loo et al. [25] formalized two-pronged designs combining multi-model agreement with gold-standard accuracy, in abstract-classification and cardiovascular domains respectively. The closest independent analog is a consensus LLM pipeline that extracted complexity-graded variables across OpenAI, Anthropic, and Google model families. Additionally, it validated them against clinician-adjudicated error in multiple sclerosis and reached expert-grade reliability through model agreement [26]. The present work differs in that it pairs four single-vendor extractions with chance-corrected, complexity-stratified agreement statistics on registry records rather than fusing them by consensus. A complementary line of evidence cautions that agreement is not accuracy: the MedEvidence benchmark showed that models can be jointly consistent yet wrong, lacking scientific skepticism [27]. Mao et al. [28] scored four frontier platforms, including Claude and Gemini, against expert-rated trial conclusions using two independent raters, a structural template close to the validation design adopted here. Methodologically, our three-tier complexity framing is mirrored by Murin [29], who identified the binary-versus-multi-way schema distinction as the largest single source of extraction disagreement, and is corroborated by accuracy studies showing numeric fields to be harder than string fields [30] and subjective tasks harder than objective ones [31]. Our use of Cohen’s and Fleiss’ kappa as a multi-model reliability signal follows Jain et al. [32], and our interpretation of the kappa paradox under high class prevalence is informed by the documented self-inconsistency of LLM judges across runs [33] and recent guidance on agreement-metric selection [34]. Against this landscape, the present study is, to our knowledge, the first to benchmark four frontier commercial and open-weight LLMs on schema-constrained extraction directly from ClinicalTrials.gov registry records in a neuromodulation (tDCS in PD) domain. With the addition of an expert gold standard, it further tested whether the systematic decline of inter-model agreement with task complexity tracked a corresponding decline in correctness.

Beyond these directly comparable studies, a broader benchmarking literature establishes that no single LLM dominates across biomedical and clinical trial extraction tasks, with different systems excelling on different tasks and datasets [35,36], motivating our second hypothesis of model-specific rather than uniform superiority. High run-to-run consistency need not imply correctness [37], so validating extractions against an expert-curated standard, not inter-model agreement alone, has been demonstrated for movement-disorder case reports and large-scale clinical pipelines [38,39], with Cohen’s kappa increasingly used to quantify LLM–human reliability [40].

Transcranial direct current stimulation (tDCS) represents an exemplary test case for evaluating LLM extraction capabilities. This non-invasive brain stimulation technique modulates cortical excitability through mechanisms involving synaptic plasticity or changes in neuronal activity [41,42]. Applied to brain areas such as the primary motor cortex (M1) [43,44], supplementary motor area (SMA) [45,46], dorsolateral prefrontal cortex (DLPFC) [47], and cerebellum [48], tDCS has shown promise as an adjunct treatment for PD. While not all studies demonstrate positive effects [49,50], the majority of the literature reports motor skill improvements averaging approximately 10–15% following tDCS applied at 1–2 mA for 20 min over one or more days [51,52]. Due to the large volume of research on tDCS for PD over the past decade and the heterogeneity in methodology employed, this represents a research area where emerging interventions could benefit from more rapid and thorough data extraction [53,54].

Despite these advances, critical gaps remain. Most published LLM benchmarks focus on structured or synthetic data (e.g., GLUE [55], SuperGLUE [56], MMLU [57]), overlooking the complexity inherent in real clinical protocols [58]. Systematic evaluations involving unstructured, protocol-level data, including stimulation parameters, anatomical targets, and multi-component interventions, remain scarce. Furthermore, conventional database searches exhibit substantial variability: identical queries on ClinicalTrials.gov yield 30 to 72 results depending on syntax variations such as apostrophe usage or Boolean operators [59], highlighting limitations of keyword-based retrieval.

What remains unclear is at what level of protocol complexity multiple LLMs begin to diverge when extracting authentic clinical trial data, a gap that matters because understanding model-specific strengths and failure modes is essential for designing reliable, scalable extraction systems.

The primary purpose of this study was to evaluate inter-model agreement among four LLMs (o4-mini-high, Claude-Sonnet-4, Gemini 2.5-Pro, and Llama-4-Maverick) for extracting clinical trial data from ClinicalTrials.gov, using tDCS trials in PD as a test case. It was hypothesized that inter-model agreement would decrease as extraction task complexity increased, with higher agreement for simple binary classifications (e.g., brain stimulation presence, invasiveness), moderate agreement for categorical extractions (e.g., stimulation type), and lower agreement for complex multi-component tasks (e.g., anatomical targets, numeric parameters). It was further hypothesized that different LLMs would demonstrate distinct strengths across task types, with no single model outperforming across all extraction categories, reflecting differences in training data, architecture, and optimization objectives. A third question concerned whether the decline in inter-model agreement reflects a corresponding decline in accuracy. The extractions were therefore validated against an expert-annotated gold standard to determine whether inter-model agreement is a reliable surrogate for correctness. To test these hypotheses, a structured JavaScript Object Notation (JSON) schema was deployed across all four models, with inter-model agreement quantified using Cohen’s Kappa and percentage agreement metrics and accuracy benchmarked against expert annotations.

## 2. Materials and Methods

### 2.1. Data Source and Collection

Clinical trial data related to PD were collected from ClinicalTrials.gov using the Application Programming Interface (API) v2 on 5 July 2025. ClinicalTrials.gov is a comprehensive registry maintained by the National Library of Medicine (NLM) at the National Institutes of Health (NIH), Bethesda, MD, USA, and provides access to information on publicly and privately supported clinical studies conducted worldwide. The data collection process utilized the following search parameters: Primary condition: “Parkinson”; search fields: both condition and title fields were queried; API endpoint: https://clinicaltrials.gov/api/v2/studies (accessed on 5 July 2025); data format: JSON.

A systematic approach for retrieving and analyzing clinical trial data was implemented using the ClinicalTrials.gov API (v2). The data collection pipeline was developed in Python 3.12 (Python Software Foundation, Wilmington, DE, USA), utilizing custom functions to interact with the API’s endpoints [60,61]. The primary data retrieval function was designed to handle large-scale data collection through paginated requests, with configurable batch sizes up to 1000 records per request to optimize retrieval efficiency while respecting API rate limits. A total of 4201 clinical trial records were retrieved and analyzed. The dataset contained comprehensive information for each clinical trial, including study identifiers (NCT IDs), study design information, intervention details, eligibility criteria, study status and timeline, location information, and outcome measures (see Appendix A for complete list of API fields). The quality and completeness of the dataset were assessed, resulting in 3707 records (88.2%) containing intervention data, 4201 records (100.0%) including study summary descriptions, and 4176 records (99.4%) reporting enrollment counts.

### 2.2. Search Strategy and Trial Selection

Using “Parkinson’s disease and tdcs” returned only 30 studies, while “Parkinson’s disease tdcs” (without “and”) yielded 55 studies (19–20 July 2025). The variation extended further when comparing possessive and non-possessive forms: “Parkinson disease tdcs” returned 62 studies, “Parkinson’s disease” as the condition and “tdcs” as the intervention returned 64 studies, while “Parkinson disease” as the condition and “tdcs” as the intervention yielded 72 studies (19–20 July 2025). This variability in search results exemplifies how the current clinical trials database interface can hinder researchers and the public from accessing timely and accurate information. These counts are reported to illustrate interface-driven variability and to motivate a systematic, reproducible retrieval strategy. They are not a formal evaluation of retrieval recall or precision, which was beyond the scope of this study.

**Figure 1:**
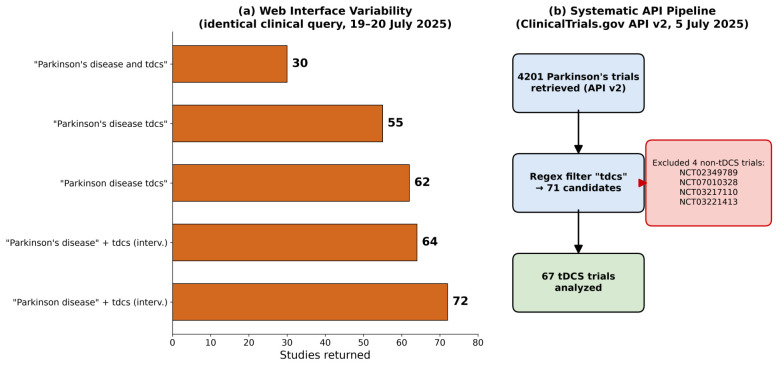
**Search strategy comparison for tDCS clinical trials in PD.** (**a**) Web interface variability (19–20 July 2025): identical clinical queries yielded 30–72 studies depending on syntax variations (apostrophes, boolean operators, field specifications), demonstrating limitations of traditional keyword searches. (**b**) Systematic API-based extraction pipeline (5 July 2025): comprehensive retrieval of 4201 Parkinson’s trials followed by regex filtering (“tdcs”) identified 71 candidates. Manual validation excluded 4 non-tDCS trials (NCT02349789, NCT07010328, NCT03217110, NCT03221413), yielding 67 trials for multi-LLM analysis.

To ensure comprehensive coverage, a case-insensitive regex filter for the term “tdcs” was applied across all retrieved record fields (including intervention name, intervention description, brief summary, and detailed description), yielding 71 trials. In routine registry practice, tDCS trials are indexed under the acronym in at least one of these fields, so the acronym-based filter captured the relevant records. Nonetheless, protocols that describe transcranial direct current stimulation only by its full name, with the acronym appearing nowhere in the record, could in principle be missed, a recall limitation revisited in the Discussion. Four trials (NCT02349789, NCT07010328, NCT03217110, and NCT03221413) were then excluded after manual review of their protocol text, as they did not employ tDCS as their intervention modality. This curation step was performed by a single analyst (R.J.Y.) without an independent second rater, so the selection decision itself was not subjected to inter-rater verification. The remaining 67 trials were selected for LLM analysis (Figure 1).

### 2.3. Data Versioning

The data management strategy employed multiple storage formats throughout the processing pipeline. Data were stored in Parquet format [62], read and written using the pyarrow library (version 7.0.0), for quick access and transactions between notebooks. Processed data were maintained in both CSV and Excel formats for accessibility. The initial stage involved extracting and storing data simultaneously in three formats: comma-separated values (CSVs) for broad compatibility, Excel for human readability, and Parquet for efficient computational processing.

### 2.4. LLM Configuration and Architecture

The analytical pipeline was implemented in Python 3.12, leveraging several key libraries for specific functionalities. The pandas library (version 1.3.0) [63] handled data transformation, numerical operations were performed using NumPy (version 1.20.0) [64], and LLM API calls were executed using the requests library (version 2.25.0) via OpenRouter’s OpenAI-compatible endpoints. Statistical analyses were carried out using scikit-learn (version 1.0.0), and all figures were produced with Matplotlib (version 3.4.0) and seaborn (version 0.11.0).

**Figure 2:**
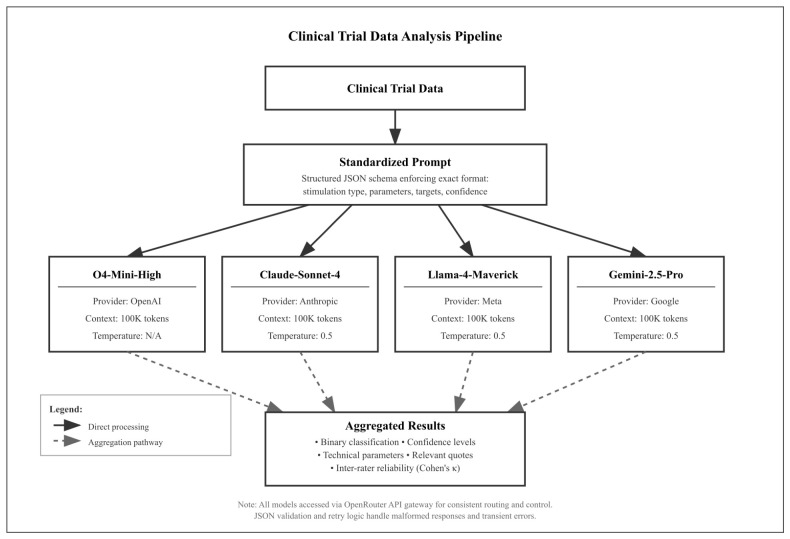
**LLM analysis workflow for clinical trial data review.** A standardized prompt was processed through four language models (o4-mini-high, Claude-Sonnet-4, Gemini-2.5 Pro, and Llama-4 Maverick), with results aggregated to produce comprehensive trial assessments. Dashed lines indicate a suggested aggregation process.

The analysis framework incorporated four LLMs accessed through the OpenRouter API infrastructure, similar to methodology used by Khan et al. [65] and Young et al. [5] (Figure 2). OpenRouter is an LLM gateway site that allows API requests to route to different models or providers, allowing for better consistency and control. The first model, o4-mini-high (OpenAI, San Francisco, CA, USA) [9], was chosen because it is OpenAI’s newer model and is frequently used in clinical applications despite being closed-source, providing a strong foundational benchmark for evaluation. O4-mini-high was configured with a maximum context window of 100,000 tokens and, as a reasoning-focused model, was queried without temperature settings. Certain models support temperature and others do not. If the model supported temperature, it was set to 0.5 [66]. The secondary model, Claude-Sonnet-4 (Anthropic, San Francisco, CA, USA), was selected due to its safety-first design philosophy and large context window of 100,000 tokens, which is beneficial for analyzing detailed clinical trial descriptions. It also utilized a temperature setting of 0.5. The third model was Google Gemini 2.5 Pro (Google, Mountain View, CA, USA) with a temperature setting of 0.5 and a max token limit of 100,000. Finally, Llama-4-Maverick Instruct (Meta, Menlo Park, CA, USA) [67] was included because it is open-source and could be embedded into clinical applications or deployed locally, thus offering an alternative architectural perspective to the analysis. A temperature of 0.5, rather than fully deterministic decoding (temperature 0), was applied to temperature-supporting models to balance reproducibility against the flexibility required to interpret heterogeneous, non-standardized protocol phrasing. O4-mini-high, as a reasoning-optimized model, does not expose a temperature parameter and was queried in its default configuration. Each model generated a single response per trial. Repeated sampling to quantify within-model output variability was not performed, a constraint addressed in the Discussion. To support reproducibility despite the rapid deprecation of commercial model versions, the exact model identifiers (openai/o4-mini-high, anthropic/claude-sonnet-4, google/gemini-2.5-pro, and meta-llama/llama-4-maverick, as routed by OpenRouter at the time of analysis) and all raw model outputs are archived in the public repository.

### 2.5. Prompt Design and JSON Schema

For each clinical trial, the DetailedDescription field (or BriefSummary when unavailable) was extracted directly from the ClinicalTrials.gov API response and passed as context to each LLM. This approach provided the model with the complete protocol description as registered, enabling determination of brain stimulation presence and associated parameters [68,69]. A clear directive was provided: “Analyze whether brain stimulation was used in this trial. If so, provide details.” This instruction explicitly informed the LLM of the required output scope (i.e., binary classification of brain stimulation usage and associated details). Careful phrasing minimized ambiguity and guided the model to generate precise, relevant information [70,71,72].

A structured JSON schema was employed to capture the following information: binary classification of whether brain stimulation was used (“brain_stimulation_used”: “Yes”, “No”, or “Unknown”); technical parameters including the primary stimulation type, an invasiveness flag (“is_noninvasive”), target brain regions, and session details; confidence level of the model’s determination (“confidence_level”: “High”, “Medium”, or “Low”); and relevant quotes grounding the model’s assertions in the source text, offering transparency into its decision-making process [73]. The complete prompt and JSON schema are reproduced in Appendix B.

The final prompt architecture was designed to ensure consistent, structured responses from LLMs, particularly when extracting information about brain stimulation in clinical trials. By implementing this architecture, unstructured variance in LLM outputs was reduced and alignment with downstream analysis requirements was ensured [74,75]. Responses that did not conform to the schema or omitted a field were recorded as missing for that field rather than imputed. Accordingly, inter-model agreement was computed pairwise over the trials for which both models returned a value. In the accuracy analysis (Section 3.6), by contrast, a field a model left unextracted or labeled “Unknown” was scored as an error against the expert value. The schema fields were chosen to span the three complexity tiers under study: a binary presence flag, categorical modality and target fields, and numeric stimulation parameters. These fields were grounded in the tDCS-in-Parkinson’s literature (current intensity, session duration, and common cortical targets such as M1, SMA, and DLPFC), and the full field set was reviewed before deployment by the study’s domain expert in transcranial brain stimulation (B.P.). A single canonical prompt and schema were issued to all four models to hold the instruction constant across systems. Consequently, no prompt-paraphrase or schema-variant sensitivity analysis was performed, and the robustness of extraction to alternative phrasings remains unquantified (see Limitations).

### 2.6. Statistical Analysis

Agreement between model pairs was quantified using Cohen’s Kappa (κ) for categorical variables, interpreted according to Landis and Koch [76]: κ < 0.00 indicates poor agreement, 0.00–0.20 slight, 0.21–0.40 fair, 0.41–0.60 moderate, 0.61–0.80 substantial, and 0.81–1.00 almost perfect agreement. Cohen’s κ was undefined for any field that is invariant across raters, where every observation falls in a single category. With no marginal variance to correct for, such fields are reported with percentage agreement and κ marked as undefined rather than as 1.00. Percentage agreement was calculated as the proportion of concordant responses. Wilson score intervals provided 95% confidence intervals (CIs) for percentage agreement and for percentage accuracy against the gold standard. Pairwise Cohen’s Kappa captures only two raters at a time, so multi-rater agreement across all four models was additionally quantified using Fleiss’ Kappa and Krippendorff’s α for categorical features and the intraclass correlation coefficient (ICC) for numeric parameters.

For numeric parameters (intensity in mA, session duration in minutes), Mean Absolute Error (MAE) and Root Mean Square Error (RMSE) quantified extraction differences between model pairs, with Pearson correlation coefficients assessing linear relationships. Numeric values were normalized to a common unit (milliamperes for intensity, minutes per session for duration) before comparison. Parameters not stated explicitly in the source text were recorded as “Not stated” and excluded from the error computation rather than imputed. The numeric metrics are therefore defined only over trials in which both compared raters (two models, or a model and the expert) reported an explicit value. Agreement patterns were visualized using confusion matrices and scatter plots.

Task complexity was operationalized using a three-tier classification: Level 1 (Simple) included binary yes/no classifications with unambiguous criteria (brain stimulation presence, invasiveness); Level 2 (Moderate) included categorical extractions from constrained option sets (primary stimulation type, confidence level); Level 3 (Complex) included multi-component extractions requiring integration of distributed information (anatomical targets, numeric parameters with unit conversion). The hypothesized decline in agreement across these tiers was characterized descriptively, by comparing mean percentage agreement at each complexity level. The pairwise agreement values are not mutually independent (each of the four models contributes to multiple pairs), so inferential tests that assume independent observations were deliberately not applied, as they would overstate the available degrees of freedom. Finally, to assess accuracy rather than concordance alone, a domain expert (B.P.), whose published research program centers on transcranial brain stimulation of the motor cortex [77,78], annotated all 67 trials from the same source text supplied to the models, yielding the expert gold-standard annotations (Supplementary File S1). To confirm these expert annotations were reproducible rather than idiosyncratic, a second annotator (R.J.Y.) independently coded a 20-trial overlap to establish inter-annotator reliability (Cohen’s κ; Supplementary File S2). Each model’s extractions were scored against this gold standard as percentage accuracy for categorical features and as mean absolute error against the expert-recorded values for numeric parameters. Anatomical targets and stimulation types were compared after normalization against a fixed lexicon (Supplementary File S3) that mapped each observed surface form to its canonical anatomical target or tDCS variant. The same lexicon was applied identically to the model outputs and the expert annotations. The annotation fields and their permitted values are specified in the codebook (Supplementary File S4). To distinguish genuine extraction differences from interchangeable wording, inter-model agreement on the free-text fields was additionally recomputed under this same normalization and compared against exact-string agreement (Section 3.6). Overall accuracy can be misleading when one class dominates, so each categorical field was additionally summarized by a macro-averaged $F_{1}$ score: the unweighted mean across classes of the per-class $F_{1}$, which is the harmonic mean of the class’s precision and recall. Reporting macro-$F_{1}$ alongside accuracy surfaces the minority-class precision and recall that an aggregate accuracy can conceal.

### 2.7. Ethical Considerations

All data analyzed in this study were obtained from ClinicalTrials.gov, a publicly accessible database. No human subjects were directly involved in this research; therefore, Institutional Review Board approval was not required. All clinical trial records are de-identified and publicly available under federal regulations. The study adhered to principles of data integrity and transparency, with all analysis code and processed datasets made available for verification.

## 3. Results

To examine whether LLM agreement varied by extraction task complexity (Hypothesis 1) and whether model-specific differences existed (Hypothesis 2), pairwise agreement analyses were conducted across all four models. These agreement results were then evaluated against an expert gold standard (Section 3.6).

### 3.1. Overall Agreement Patterns

**Figure 3:**
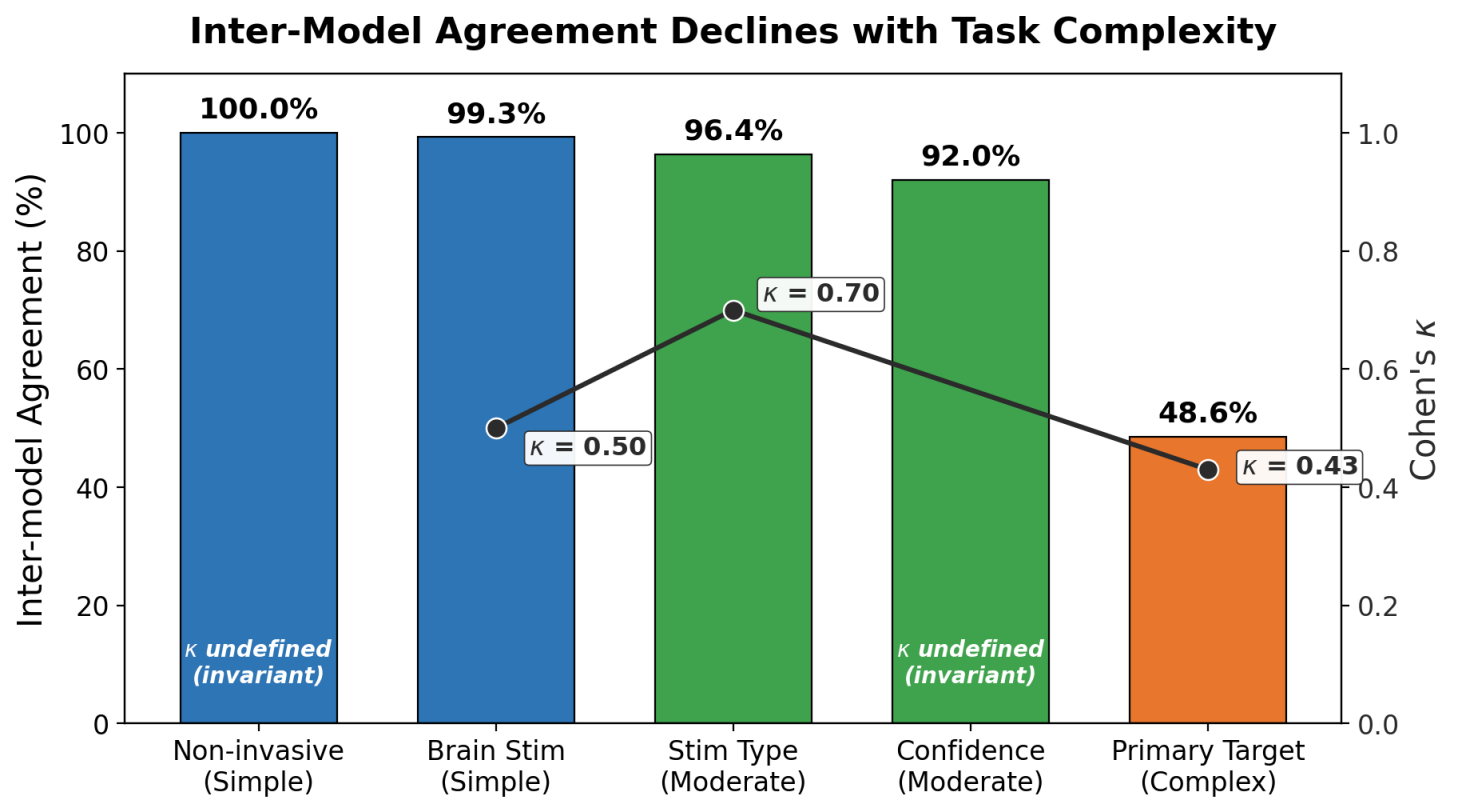
**Inter-model agreement patterns across task complexity levels.** Agreement percentages (bars) and Cohen’s Kappa values (line) demonstrate systematic decline from simple binary classifications (99–100%) through moderate categorical tasks (92–96%) to complex anatomical extractions (49%). This pattern supports Hypothesis 1: LLM agreement decreases with increasing task complexity.

Table 1 and Figure 3 summarize inter-model agreement, computed under exact-string matching, across extraction tasks, organized by complexity level. Binary classifications achieved the highest agreement: non-invasive classification reached 100% agreement (Cohen’s κ undefined, every model that returned a value returned “non-invasive”, so the field is invariant and κ has no negative class to correct for; 95% CI for percentage agreement: 94.4–100.0%), while brain stimulation detection achieved 99.3% agreement (κ = 0.50, 95% CI: 92.0–100.0%) (the disparity between high percentage agreement (99.3%) and moderate κ (0.50) for brain stimulation detection reflects the well-documented kappa paradox under high class prevalence: when one class dominates (66 of 67 trials classified “Yes”), a single discordant observation disproportionately reduces κ even when raw concordance remains near-perfect [79]). Categorical extraction of primary stimulation type maintained substantial agreement at 96.4% (κ = 0.70, 95% CI: 89.3–99.1%). Models’ self-reported confidence assessments agreed at 92.0% (Table 1). Since this field reflects each model’s internal certainty in its own determination rather than a substantive clinical extraction, only percentage agreement was reported and Cohen’s κ was marked not applicable. Complex anatomical target extraction showed markedly lower agreement at 48.6% (κ = 0.43, 95% CI: 36.7–60.7%). As the gold-standard validation establishes (Section 3.6), this exact-string value rose to 93.9% on the same named-target trials once anatomical synonyms (e.g., “M1” and “primary motor cortex”) were treated as equivalent. The low figure reflected interchangeable wording rather than disagreement about the underlying target.

Multi-rater agreement across all four models corroborated the pairwise pattern: Fleiss’ Kappa and Krippendorff’s α agreed closely on every feature (0.66/0.66 for brain stimulation presence, 0.70/0.69 for primary stimulation type, 0.43/0.43 for primary anatomical target, and 0.59/0.59 for secondary anatomical target; non-invasive classification was invariant across all models and is therefore undefined). For numeric parameters, the intraclass correlation coefficient indicated substantial average-measures agreement for session duration (${\mathrm{ICC}}_{2,k}$ = 0.70, *n* = 19) and perfect agreement for stimulation intensity (ICC=1.00, *n* = 8 trials with a parseable value from both compared models; per-model coverage against the expert was 8–10). Descriptively, mean pairwise agreement declined across the three complexity tiers: 99.3% and 100% on the simple binary fields (brain stimulation presence and non-invasiveness), 96.4% and 92.0% on the moderate categorical fields (stimulation type and confidence), and 48.6% on the complex anatomical target under exact-string matching. These tier-level values are derived from non-independent pairwise comparisons among only four models (each model contributing to multiple pairs), so the gradient was summarized descriptively rather than with an inferential test. Such a test would treat the correlated pairwise observations as independent and overstate the available degrees of freedom. The pairwise and multi-rater analyses nonetheless converge on the same pattern: inter-model agreement declines steeply as extraction complexity increases.

### 3.2. Brain Stimulation Detection

**Figure 4:**
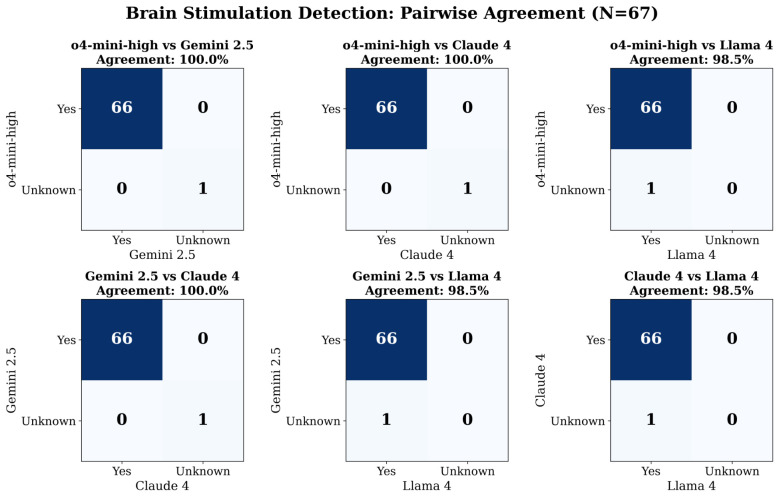
**Pairwise confusion matrices for brain stimulation detection.** Each matrix shows agreement between a model pair for the Yes/Unknown classification (N = 67 trials), with per-pair percentage agreement reported above each panel. O4-mini-high, Gemini-2.5, and Claude-4 demonstrate perfect agreement (100%, diagonal dominance), while every comparison involving Llama-4-Maverick shows 98.5% agreement, owing to the single trial (NCT01113086) that Llama-4-Maverick labeled “Yes” while the other three models labeled “Unknown”. Non-invasive classification was concordant for all model pairs (100% agreement; κ undefined, as the field is invariant). Numbers represent trial counts; color intensity reflects frequency.

For binary brain stimulation detection, all six model pairs achieved at least 98.5% agreement (Figure 4). O4-mini-high, Gemini-2.5, and Claude-4 produced identical classifications, each labeling a single trial (NCT01113086) “Unknown” and the remaining 66 “Yes”, so the three comparisons among them agreed perfectly. Llama-4-Maverick instead returned “Yes” for all 67 trials, so each comparison involving Llama-4 differed on that one trial (98.5% agreement). With one class so heavily dominating, the overall Cohen’s κ fell to 0.50 despite 99.3% mean agreement (Table 1), the kappa paradox. Detecting whether brain stimulation was used is thus near-perfectly concordant across models. However, as with non-invasive classification, this near-invariance means agreement alone establishes little, and accuracy must be judged against the gold standard (Section 3.6).

### 3.3. Non-Invasive Classification

All model pairs achieved 100% agreement on non-invasive classification: across the 65 trials with a valid extraction from both models, every comparison concordantly returned “non-invasive.” Because no model ever produced the minority (“invasive”) label, this field is invariant and Cohen’s κ is undefined. The 100% raw agreement reflects the unambiguous framing of tDCS as non-invasive. As the gold-standard validation shows (Section 3.6), however, this very unanimity masks the one genuinely invasive protocol that every model misclassified, a concrete instance of the shared-error failure mode that agreement cannot detect.

### 3.4. Numeric Parameter Extraction

**Figure 5:**
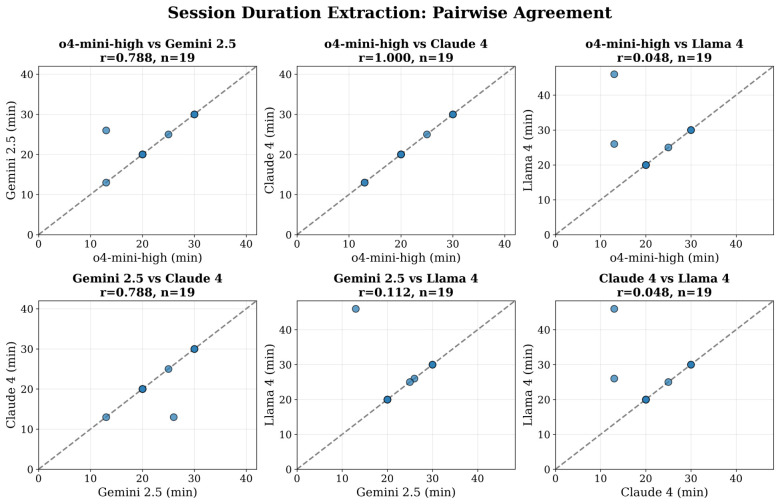
**Duration extraction scatter plots across model pairs.** Each panel shows pairwise comparison of extracted session durations (minutes). O4-mini-high vs Claude-4 (top center) demonstrates perfect agreement (*r* = 1.000, points on the identity line). Comparisons involving Llama-4-Maverick (top right and bottom row) show marked divergence (*r* < 0.12), with extracted values largely independent of other models. Dashed lines indicate perfect agreement (y = x).

Table 2 and Figure 5 show the duration extraction agreement metrics. O4-mini-high and Claude-4 demonstrated perfect agreement (MAE = 0.00, RMSE = 0.00, *r* = 1.000), suggesting shared parsing algorithms for temporal expressions. In contrast, Llama-4-Maverick showed systematic divergence from other models (MAE = 1.74–2.42, *r* = 0.05–0.11), with extracted duration values largely independent of other models’ outputs. Session duration was reported in extractable, comparable form for only 19 of the 67 trials, and stimulation intensity for only 8. The remaining protocols did not state these parameters in their registry text. Pairwise numeric metrics were therefore computed only over trials in which both compared models returned a parseable value, and the corresponding model-specific conclusions should be interpreted in light of these small samples. In short, numeric parameters reveal the clearest model-specific split (o4-mini-high and Claude-Sonnet-4 align exactly while Llama-4-Maverick diverges) and are disclosed in only a minority of registry records.

### 3.5. Model Agreement Summary

**Figure 6:**
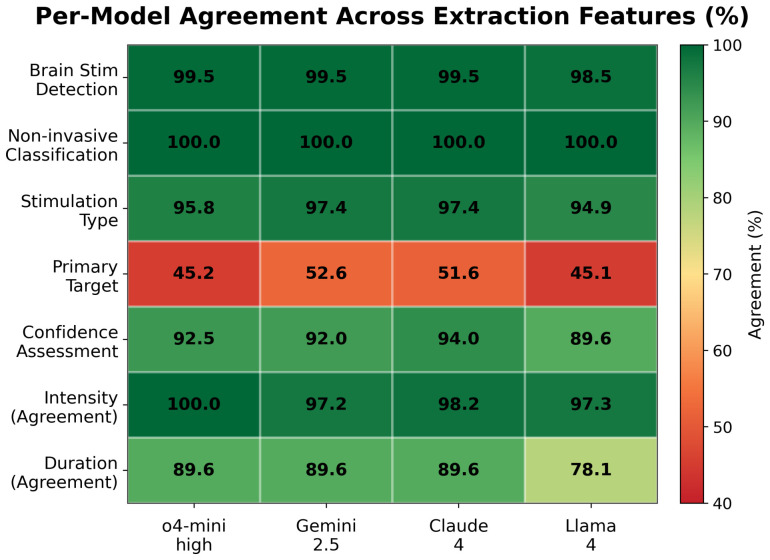
**Model agreement heatmap across extraction features.** Color encodes agreement percentage (green = higher, red = lower). All models achieve near-ceiling agreement on binary classifications (top rows) but show marked divergence on duration extraction (bottom row), with Llama-4-Maverick demonstrating systematic differences from closed-source models.

Figure 6 presents an agreement heatmap summarizing model-specific strengths across all evaluated features. All models achieved greater than 98% agreement on brain stimulation detection and 100% on non-invasive classification. For stimulation type extraction, Gemini-2.5 and Claude-4 led at 97.4%, followed by O4-mini-high (95.8%) and Llama-4 (94.9%). Primary target extraction showed the greatest variability, with Gemini-2.5 highest (52.6%) and Llama-4 lowest (45.1%). Secondary anatomical targets were specified in only 9 of 67 trials, as protocols overwhelmingly named a single target. Within this small subset, inter-model agreement fell to the lowest range of any extraction task, comparable to or below primary-target agreement. Given the limited sample (n = 9), this is interpreted cautiously, but it reinforces that distributed, secondary anatomical detail is among the least reliably extracted elements and a clear candidate for human validation. Duration agreement patterns revealed the starkest model-specific differences, with O4-mini-high and Claude-4 achieving near-perfect agreement while Llama-4 diverged substantially.

In summary, inter-model agreement exceeded 95% for binary classifications, maintained substantial agreement for categorical extractions (κ = 0.70), and declined to below 50% for complex anatomical targets, a pattern consistent with Hypothesis 1 predictions regarding task complexity effects. Regarding Hypothesis 2, distinct model-specific strengths emerged: o4-mini-high and Claude-Sonnet-4 demonstrated perfect agreement on numeric parameters, Gemini 2.5-Pro showed balanced agreement across categories, and Llama-4-Maverick provided consistent categorical classification but diverged on duration extraction.

### 3.6. Validation Against an Expert Gold Standard

To test whether the observed decline in inter-model agreement reflects a true decline in accuracy, a domain expert (B.P.) independently annotated all 67 trials from the same registry text supplied to the models, yielding a gold standard against which each model’s extractions were scored. A second annotator (R.J.Y.) independently coded a 20-trial overlap to establish inter-annotator reliability. The two annotators agreed on every categorical field, and on every numeric value once intensities and durations were normalized to a common unit. Accordingly, Cohen’s κ was 1.00 for the fields that varied within the overlap (non-invasive classification and anatomical target) and was undefined for the fields that were invariant within the overlap (brain stimulation presence and stimulation type), where unanimous agreement leaves no marginal variance to correct for. This confirms a reliable reference standard. Anatomical targets were scored after synonym normalization (e.g., “M1” and “primary motor cortex” were treated as equivalent) and stimulation types after tDCS-variant normalization. The full normalization lexicon (every surface form mapped to its canonical target or stimulation type) is provided as Supplementary File S3 and in the project repository, and numeric values were parsed to a common unit (milliamperes for stimulation intensity and minutes per session for duration). Annotators were study co-authors rather than blinded external raters, so this reliability estimate should be read as an upper bound (see Limitations).

Scored over all 67 trials, with a field that a model left unextracted or labeled “Unknown” counted as an error, all four models extracted with high accuracy across every categorical feature (Table 3): mean accuracy was 98.9% (95% Wilson CI 97–100) for brain stimulation presence, 96.6% (94–98) for non-invasive classification, 93.7% (90–96) for stimulation type, and 95.5% (92–97) for the primary anatomical target. The small per-model spread on the binary fields reflects these differing numbers of unextracted values rather than additional substantive errors. The only substantive non-invasive error was the single invasive protocol that all four models misclassified. Overall accuracy can be inflated when one class dominates, so the macro-averaged $F_{1}$ score, which weights every class equally, was also reported. Macro-$F_{1}$ was high for the anatomical target (0.85), but was substantially lower for non-invasive classification (0.49) and stimulation type (0.57). High accuracy on these fields is carried by the majority class and conceals weak performance on the rare minority classes (the single invasive protocol; the few non-tDCS modalities that appear in trials combining tDCS with another stimulation arm). This is the accuracy paradox, a caution that accuracy alone is an inadequate metric on the degenerate, pre-filtered fields. For numeric parameters, o4-mini-high and Claude-Sonnet-4 reproduced the expert-recorded session durations exactly (MAE = 0.00, *n* = 19), while Llama-4-Maverick diverged (MAE = 2.42). Stimulation intensity was recovered with near-perfect accuracy by all models (MAE ≤ 0.15, *n* = 8–10).

**Figure 7:**
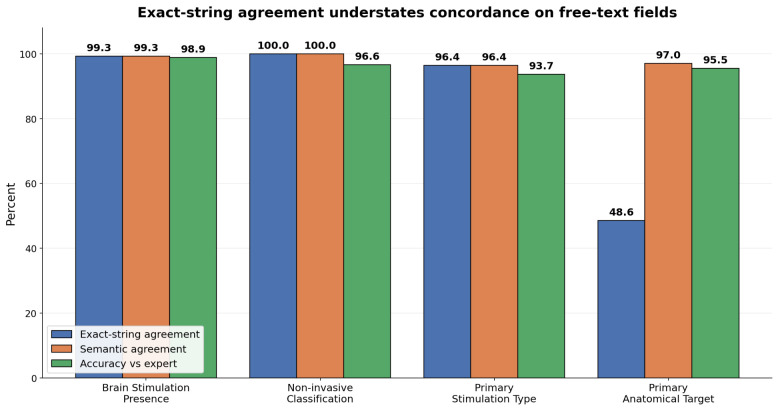
**Exact-string agreement understates concordance on free-text fields.** For each extraction feature, inter-model agreement under exact-string matching (blue) and under semantic matching (orange) is compared with accuracy against the expert gold standard (green). The three coincide for binary and categorical tasks. For the primary anatomical target, exact-string agreement is only 48.6%, but semantic agreement (97.0%) and expert accuracy (95.5%) are both high and nearly identical, showing that the apparent agreement–accuracy gap is an artifact of the scoring criterion, not a divergence between concordance and correctness.

Critically, this validation resolves an apparent paradox in the agreement data. For the primary anatomical target, exact-string inter-model agreement was only 48.6% (Table 1), yet accuracy against the expert reached 94–97%. This is not evidence that agreement diverges from accuracy. It is an artifact of the matching criterion. The 48.6% figure counts “M1” and “primary motor cortex” as a disagreement, whereas the accuracy analysis treats them as equivalent. When the same synonym normalization is applied to both analyses, inter-model agreement on the 33 named-target trials rises from 48.6% to 93.9%, closely tracking the 90.9% mean accuracy on that subset, and across all 67 trials, semantically normalized agreement reaches 97.0% against 95.5% accuracy (Figure 7). Agreement and accuracy therefore coincide once they are measured on a common basis. This convergence is not an artifact of shared normalization: inter-model agreement (model against model) and gold-standard accuracy (model against expert) are structurally distinct comparisons. Furthermore, a common lexicon constrains them only at the level of surface form, leaving each free to diverge on substantive disagreement. Therefore, their alignment reflects concordant content instead of a scoring identity. The residual shared errors that agreement alone cannot detect are exemplified by the invasive protocol misclassified by all four models. This confirms that the two measures retain independent information. The implication is methodological: free-text clinical fields must be evaluated by semantic equivalence rather than string identity, or inter-model agreement will systematically understate both concordance and correctness. The earlier interpretation of low exact-match agreement as evidence of unreliable complex-field extraction was, in this light, an overstatement.

The principal residual risk is therefore not low accuracy but *shared* error, the failure mode that inter-model agreement cannot, by construction, detect. Such errors were rare but consequential. In trial NCT06505460, which paired non-invasive tDCS and magnetic resonance-guided focused ultrasound (MRgFUS) with invasive deep brain stimulation (DBS), all four models classified the protocol as non-invasive. Accordingly, each cited the exact sentence that named the DBS arm, yet still overlooked it, whereas the expert correctly flagged the trial as invasive on the basis of that surgically implanted component. This single case is precisely why the non-invasive field, despite 96.6% accuracy, has a macro-$F_{1}$ of only 0.49. Every model missed the one invasive protocol, so that class scores zero recall (0 of 1) and a zero $F_{1}$ (with no model ever predicting “invasive,” its precision is undefined). Macro-$F_{1}$ weights the two classes equally, so averaging that zero against the near-perfect non-invasive class (about 0.98) halves the score to roughly 0.49. With only one invasive trial in the corpus, this recall figure is itself imprecise, but it concretely illustrates the hazard: unanimous concordance offered no protection against an error common to all systems. In summary, expert validation establishes that these models achieve high accuracy on the majority of fields, that overall accuracy can nonetheless mask poor minority-class performance, and that the residual risk warranting targeted human oversight is the rare shared error rather than broad unreliability on complex fields.

## 4. Discussion

This study evaluated inter-model agreement among four LLMs extracting clinical trial data from tDCS trials in PD. The primary finding was that LLM agreement decreased systematically with task complexity: near-perfect agreement (greater than 99%) was observed for binary classifications, substantial agreement (κ = 0.70) for categorical extractions, and markedly lower concordance (48.6%, κ = 0.43) for complex anatomical targets, consistent with the first hypothesis. Supporting the second hypothesis, distinct model-specific strengths emerged rather than uniform superiority: o4-mini-high and Claude-Sonnet-4 excelled at numeric precision, Gemini 2.5-Pro demonstrated balanced agreement, and Llama-4-Maverick provided consistent categorical classification despite divergence on temporal parameters. Collectively, these findings establish that multi-model LLM architectures extract clinical trial data accurately across task tiers, that low inter-model agreement on complex fields signals surface-form variation rather than poor accuracy, and that targeted human oversight is warranted chiefly to catch the rare error shared across models, a critical insight for designing scalable clinical data extraction systems.

The systematic decline in exact-string agreement across complexity tiers (Figure 3) likely reflects the increasing semantic ambiguity and contextual dependency of complex protocol descriptions, where the relevant information is distributed across multiple text segments requiring integration. Critically, this decline is a property of exact-string scoring rather than of extraction reliability. As the validation shows (Section 3.6), semantic matching closes most of the gap, so the complexity gradient describes surface-form concordance rather than correctness. This pattern was not an artifact of our data or design, because an independent evaluation of LLM extraction from discharge summaries, scored under exact, fuzzy, and semantic (LLM-judge) matching, reported a consistent 40% to 50% exact-versus-semantic gap across prompting, retrieval-augmented generation, and supervised fine-tuning [80]. These results mirror our own exact-string (48.6%) versus semantically matched (93.9% to 97.0%) agreement. Finally, its persistence across such different strategies established the gap as a property of string-based scoring itself and not of any specific model or method. These considerations support and confirm the view that free-text clinical fields must be judged by semantic equivalence before low agreement is read as unreliability. These findings align with Lai et al. [22] and Liu et al. [23], who reported varying LLM concordance in clinical extraction tasks. To our knowledge, this study provides one of the first systematic characterizations of how LLM agreement relates to extraction task complexity using real clinical trial protocols.

Confusion matrices (Figure 4) and scatter plots (Figure 5) revealed distinct model clustering patterns. O4-mini-high and Claude-Sonnet-4 demonstrated perfect agreement (*r* = 1.000) on duration extraction, suggesting shared parsing algorithms or training approaches. In contrast, Llama-4-Maverick showed systematic divergence (*r* < 0.12), with extracted duration values largely independent of other models’ outputs. This pattern suggests that Llama-4-Maverick employs fundamentally different parsing rules for temporal expressions, particularly for complex duration specifications such as multi-session protocols. The model-specific strengths observed, with closed-source models excelling at numeric precision and open-source alternatives providing consistent categorical classification, support ensemble strategies that leverage complementary capabilities. This closed- versus open-weight divide plausibly reflects differences in post-training rather than raw capability: the proprietary reasoning and instruction-tuning pipelines of o4-mini-high and Claude-Sonnet-4 may more strictly enforce the numeric formatting and unit normalization that exact duration matching rewards, whereas the open-weight Llama-4-Maverick, optimized for broad generative competence, applies less consistent temporal parsing. This interpretation is necessarily tentative, as the training corpora and procedures behind the commercial models are not publicly disclosed.

The agreement heatmap (Figure 6) summarizes model strengths across all evaluated features, revealing consistently high agreement for categorical tasks but marked divergence for duration extraction and anatomical targeting. These patterns have practical implications for the data extraction step of evidence synthesis specifically, not for the systematic review process as a whole, which also entails comprehensive search, screening, risk-of-bias appraisal, and qualitative synthesis that this study did not address. Restricted to that step, the pipeline processed 67 trials across four models in under 24 h, against the considerable manual effort that structured extraction can require [81]. The contribution is therefore framed as accelerating one labor-intensive component of evidence synthesis rather than compressing an entire review. Crucially, expert validation tempered this interpretation: accuracy remained high (mean 93.7–98.9%) even for the complex anatomical-target field where inter-model agreement fell below 50%, indicating that low agreement chiefly reflects interchangeable wording and the many trials with no target to extract, rather than widespread error. Inter-model agreement therefore functions as a conservative screening signal. The failure mode that genuinely warrants human oversight is the rare error shared across all models, exemplified by the trial that combined non-invasive tDCS and MRgFUS with invasive deep brain stimulation, which every model misclassified as non-invasive. The optimal approach is thus a hybrid human-in-the-loop methodology in which expert review is concentrated on detecting such shared errors rather than on re-extracting every complex field. This same logic underpins recent work in which disagreement among four locally hosted LLMs not only triaged uncertain structured extractions, but also surfaced errors in the human reference standard itself [82]. This reinforces the idea that inter-model concordance is best used to direct expert verification rather than to replace it.

Several limitations warrant consideration. First, the focus on tDCS trials in PD (N = 67) may limit generalizability. However, the extraction methodology is domain-agnostic and adaptable to other therapeutic areas [83,84]. Second, the complexity metric employed may not fully capture the multidimensional nature of clinical trial designs [85,86,87]. Third, reliance on specific API endpoints introduces potential instability as external systems evolve [88,89]. Fourth, current LLM context window limitations could constrain analysis of increasingly complex protocols, though advances such as million-token context windows show promise [90,91]. Fifth, a single fixed prompt was used and prompt sensitivity was not systematically assessed. Each model was queried once per trial, so within-model output variability was not characterized, and high inter-model agreement could in principle be partially inflated by stochastic self-consistency [33]. Sixth, although the expert gold standard demonstrated high accuracy, it rests on a single expert annotator who is a study co-author rather than an independent, blinded external rater (with a dual-coded subset establishing reliability) within a single therapeutic domain, was scored against registry text rather than full published protocols, and relied on a synonym-normalization scheme for anatomical targets. The numeric accuracy estimates also rest on small subsets (*n* = 19 for duration, *n* = 8–10 for intensity). Seventh, the three-tier complexity scheme is an a priori operationalization rather than an externally validated construct. Eighth, the extraction schema captures only a subset of dose-defining stimulation parameters. It records modality, target, intensity, and duration but not montage, electrode polarity and placement, sham configuration, number of sessions, or cumulative dose, so the present results speak to those four fields rather than to complete protocol characterization. Ninth, because the corpus was pre-filtered to tDCS trials, the binary fields (stimulation presence and non-invasiveness) are near-degenerate, with almost every trial sharing the same label. High agreement and accuracy on these fields are therefore uninformative about discriminative ability, as no negative or non-tDCS controls were included, and trial selection itself relied on an acronym-only (“tdcs”) filter and a single-analyst exclusion step without independent adjudication. Tenth, the trial records are drawn from a public registry that may overlap with the models’ pretraining corpora, so performance may partly reflect memorization rather than reading comprehension. Accuracy is best interpreted as fidelity to the registry text rather than as evidence of generalization to unseen protocols. Despite these limitations, the systematic comparison across four state-of-the-art models using authentic clinical data provides robust evidence for the observed patterns.

The clinical context warrants explicit caution. LLM outputs can be fluent yet incorrect, so deploying automated extraction in evidence synthesis or regulatory workflows without human verification risks propagating errors into downstream clinical decisions. Accordingly, the present results support automation only for the simplest, highest-agreement tasks and mandate expert oversight for complex protocol elements. In the interest of transparency, one author (R.J.Y.) is employed by a healthcare organization and has previously received unrelated research funding from OpenAI, whose o4-mini-high model is among those evaluated. To mitigate any potential bias, all model outputs, analysis code, and the evaluation pipeline are publicly available; no model was tuned or selected on the basis of its performance; and all conclusions derive from pre-specified agreement metrics applied uniformly across the four systems.

These findings extend prior LLM benchmarking studies, which have predominantly focused on structured or synthetic data, by demonstrating agreement patterns on authentic clinical protocols. The systematic degradation of agreement with task complexity provides actionable guidance for pipeline design: simple classifications are strong candidates for automation with human audit, and even complex extractions achieve high accuracy against an expert standard, though targeted human-in-the-loop review remains valuable for detecting the rare error shared across models [92,93,94,95]. This framework directly supports emerging strategies including modular extraction pipelines [96,97], multi-agent architectures with specialized task agents [98,99,100,101], and privacy-preserving local deployments [94]. The evidence supports transitioning from single-model approaches toward ensemble or multi-model strategies that leverage model-specific strengths, particularly combining closed-source precision with open-source accessibility.

These findings also speak to the rapidly growing use of LLMs in real-world evidence synthesis, spanning human-in-the-loop and second-reviewer extraction, end-to-end and agentic systematic review automation, and living reviews [21,102,103,104,105,106,107], where comparative evaluations consistently find these systems promising but not yet ready for unsupervised use [108,109,110], reinforcing the human oversight our complexity-stratified results help delineate. In parallel, LLMs are now routinely applied to ClinicalTrials.gov protocol content and registry-scale extraction [111,112,113], and open-source, model-agnostic tooling with explicit governance and quality-control frameworks is emerging to operationalize such pipelines [114,115], the deployment context into which validated, multi-model extraction must ultimately fit.

## 5. Conclusions

To our knowledge, this study is the first to pair current-generation frontier models with a complexity-stratified, expert-validated comparison of inter-model agreement and accuracy for tDCS in PD. The main findings were that four frontier LLMs extract clinical trial data with high accuracy (mean 93.7–98.9% against an expert gold standard), and that the decline in exact-string inter-model agreement with task complexity reflects interchangeable free-text wording rather than reduced accuracy. Under semantic matching, agreement and accuracy align closely (97.0% agreement versus 95.5% accuracy for anatomical targets). The residual risk that inter-model concordance cannot detect is the error shared across all models, a failure mode our data illustrate with a single trial that all four misclassified. This is a known risk of current LLM-based data extraction workflows that can be anticipated, but at present it cannot be fully quantified or mitigated. Therefore, human–AI workflows are most valuable when expert oversight is targeted at shared-error detection. Future research should examine: (1) multi-agent architectures with specialized task agents for numeric, anatomical, and temporal extraction; (2) integration of supplementary data sources such as NIH Reporter and referenced publications; and (3) scaling validation to high-volume domains such as oncology with greater than 100,000 registered trials. As LLMs increasingly integrate into clinical research, attention to ethical implications including human oversight, transparency, and workforce considerations remains essential. Ultimately, this work advances understanding of LLM capabilities and limitations in clinical contexts, providing a roadmap for deploying artificial intelligence to accelerate evidence-based medicine.

## Figures and Tables

**Table 1:** Overall agreement summary across extraction tasks by complexity level. Cohen’s κ is reported as undefined (undef.) for the non-invasive field, which is invariant across all model outputs (every observation falls in a single category, leaving no marginal variance to correct for); percentage agreement remains fully informative.

Complexity	Feature	Agreement (%)	95% CI	Cohen’s κ
Simple	Non-invasive Classification	100.0	94.4–100.0	undef.
Simple	Brain Stimulation Presence	99.3	92.0–100.0	0.50
Moderate	Primary Stimulation Type	96.4	89.3–99.1	0.70
Moderate	Confidence Assessments	92.0	83.2–96.5	n/a
Complex	Primary Stimulation Target	48.6	36.7–60.7	0.43

**Table 2:** Duration extraction agreement metrics between model pairs.

Model Pair	MAE	RMSE	Correlation	*n*
O4-Mini vs. Gemini-2.5	0.684	2.982	0.788	19
O4-Mini vs. Claude-4	0.000	0.000	1.000	19
O4-Mini vs. Llama-4	2.421	8.137	0.048	19
Gemini-2.5 vs. Claude-4	0.684	2.982	0.788	19
Gemini-2.5 vs. Llama-4	1.737	7.571	0.112	19
Claude-4 vs. Llama-4	2.421	8.137	0.048	19

**Table 3:** Per-model extraction accuracy (%) against the expert gold standard, scored over all 67 trials (a field that a model left unextracted or returned in non-conforming form is counted as an error). Mean accuracy is shown with a 95% Wilson confidence interval; macro-averaged $F_{1}$ weights each class equally and exposes the minority-class precision and recall that overall accuracy obscures. Anatomical-target accuracy uses synonym normalization and stimulation-type accuracy uses tDCS-variant normalization; brain stimulation presence has no negative instances, as all 67 trials employ stimulation.

Feature	o4-mini	Gemini	Claude	Llama	Mean Acc. [95% CI]	Macro-$F_{1}$
Brain Stimulation Presence	98.5	98.5	98.5	100.0	98.9 [97–100]	0.99
Non-invasive Classification	95.5	95.5	97.0	98.5	96.6 [94–98]	0.49
Primary Stimulation Type	91.0	94.0	95.5	94.0	93.7 [90–96]	0.57
Primary Anatomical Target	95.5	97.0	94.0	95.5	95.5 [92–97]	0.85

## Data Availability

All clinical trial data are publicly available from ClinicalTrials.gov. Processed datasets, analysis code, and the complete extraction pipeline are available at: https://github.com/ricyoung/ClinicalTrialLLM-Extractor (accessed on 27 June 2026) (Appendix C). The normalization lexicon, gold-standard annotations, and annotation codebook are also provided as Supplementary Materials (Files S1–S4).

## Supplementary Materials

The following supporting information can be downloaded at: https://www.mdpi.com/article/10.3390/bioengineering13070773/s1, File S1: the expert gold-standard annotations for all 67 trials; File S2: the 20-trial overlap used to establish inter-annotator reliability; File S3: the anatomical synonym and tDCS-variant normalization lexicon applied in the semantic-matching analysis; File S4: the annotation codebook.

## Abbreviations

The following abbreviations are used in this manuscript:

API	Application Programming Interface
CI	Confidence Interval
DBS	Deep Brain Stimulation
DLPFC	Dorsolateral Prefrontal Cortex
F1	F1 Score (harmonic mean of precision and recall)
ICC	Intraclass Correlation Coefficient
JSON	JavaScript Object Notation
LLM	Large Language Model
M1	Primary Motor Cortex
MAE	Mean Absolute Error
MRgFUS	Magnetic Resonance-Guided Focused Ultrasound
NCT	National Clinical Trial (identifier)
NIH	National Institutes of Health
NLM	National Library of Medicine
PD	Parkinson’s Disease
RMSE	Root Mean Square Error
SMA	Supplementary Motor Area
tDCS	Transcranial Direct Current Stimulation

## Appendix A. API Fields Retrieved from ClinicalTrials.gov

The following fields were retrieved from the ClinicalTrials.gov API v2 for each clinical trial record:

NCTId, LeadSponsorClass, LeadSponsorName, Condition, OfficialTitle, BriefTitle, Acronym, StudyType, InterventionType, InterventionName, InterventionOtherName, InterventionDescription, Phase, StudyFirstSubmitDate, LastUpdateSubmitDate, CompletionDate, OverallStatus, BriefSummary, IsFDARegulatedDevice, StartDate, DetailedDescription, ConditionMeshTerm, PrimaryOutcomeDescription, SecondaryOutcomeDescription, EnrollmentCount, EnrollmentType, BaselineCategoryTitle, BaselinePopulationDescription, BaselineTypeUnitsAnalyzed, OtherOutcomeDescription, EligibilityCriteria, StudyPopulation, HealthyVolunteers, ReferencePMID, LocationCountry, PrimaryOutcomeTimeFrame, BaselineMeasureTitle, BaselineMeasureUnitOfMeasure, BaselineMeasurementValue.

## Appendix B. Extraction Prompt and JSON Schema

For each trial, the DetailedDescription field (or BriefSummary when unavailable) was supplied to every model together with the verbatim directive: “Analyze whether brain stimulation was used in this trial. If so, provide details.” Models were instructed to return a structured JSON object with the following fields:

brain_stimulation_used (“Yes”, “No”, or “Unknown”); confidence_level (“High”, “Medium”, or “Low”); stimulation_details, comprising primary_type, is_noninvasive, primary_target, secondary_targets, and stimulation_parameters (intensity, duration); and relevant_quotes grounding each determination in the source text.

The identical prompt and schema were sent verbatim to all four models through the OpenRouter API; the complete prompt and schema definition are also archived in the public repository.

## Appendix C. Code Repository

All code, data, and analysis pipelines are publicly available at:

https://github.com/ricyoung/ClinicalTrialLLM-Extractor (accessed on 27 June 2026).

## References

[1] Ricco J.B., Guetarni F., Kolh P. (2020). Learning from Artificial Intelligence and Big Data in Health Care. Eur. J. Vasc. Endovasc. Surg..

[2] Zarin D.A., Tse T., Williams R.J., Carr S. (2016). Trial Reporting in ClinicalTrials.gov—The Final Rule. N. Engl. J. Med..

[3] Zarin D.A., Fain K.M., Dobbins H.D., Tse T., Williams R.J. (2019). 10-Year Update on Study Results Submitted to ClinicalTrials.gov. N. Engl. J. Med..

[4] Chaturvedi N., Mehrotra B., Kumari S., Gupta S., Subramanya H.S., Saberwal G. (2019). Some data quality issues at ClinicalTrials.gov. Trials.

[5] Young R.J., Matthews A.M., Poston B. (2025). Benchmarking Multiple Large Language Models for Automated Clinical Trial Data Extraction in Aging Research. Algorithms.

[6] Marshall I.J., Noel-Storr A., Kuiper J., Thomas J., Wallace B.C. (2018). Machine learning for identifying Randomized Controlled Trials: An evaluation and practitioner’s guide. Res. Synth. Methods.

[7] Tsafnat G., Glasziou P., Choong M.K., Dunn A., Galgani F., Coiera E. (2014). Systematic review automation technologies. Syst. Rev..

[8] Jonnalagadda S., Petitti D. (2013). A new iterative method to reduce workload in systematic review process. Int. J. Comput. Biol. Drug Des..

[9] OpenAI (2025). Introducing OpenAI o3 and o4-mini. https://openai.com/index/introducing-o3-and-o4-mini/.

[10] Kurokawa R., Ohizumi Y., Kanzawa J., Kurokawa M., Sonoda Y., Nakamura Y., Kiguchi T., Gonoi W., Abe O. (2024). Diagnostic performances of Claude 3 Opus and Claude 3.5 Sonnet from patient history and key images in Radiology’s “Diagnosis Please” cases. Jpn. J. Radiol..

[11] Google Team (2025). Gemini 2.5: Pushing the Frontier with Advanced Reasoning, Multimodality, Long Context, and Next Generation Agentic Capabilities. arXiv.

[12] Meta (2024). Llama-Models. Official Release Announcement; No Peer-Reviewed Specification Paper Is Currently Available. https://github.com/meta-llama/llama-models.

[13] Koller D., Beam A., Manrai A., Ashley E., Liu X., Gichoya J., Holmes C., Zou J., Dagan N., Wong T.Y. (2024). Why We Support and Encourage the Use of Large Language Models in NEJM AI Submissions. NEJM AI.

[14] Alkaissi H., McFarlane S.I. (2023). Artificial Hallucinations in ChatGPT: Implications in Scientific Writing. Cureus.

[15] Tonmoy S.M.T.I., Zaman S.M.M., Jain V., Rani A., Rawte V., Chadha A., Das A. (2024). A Comprehensive Survey of Hallucination Mitigation Techniques in Large Language Models. arXiv.

[16] Gartlehner G., Kahwati L., Hilscher R., Thomas I., Kugley S., Crotty K., Viswanathan M., Nussbaumer-Streit B., Booth G., Erskine N. (2024). Data Extraction for Evidence Synthesis Using a Large Language Model: A Proof-of-Concept Study. Res. Synth. Methods.

[17] Sheikhalishahi S., Miotto R., Dudley J.T., Lavelli A., Rinaldi F., Osmani V. (2019). Natural Language Processing of Clinical Notes on Chronic Diseases: Systematic Review. JMIR Med. Inform..

[18] Stuhlmiller T.J., Rabe A., Rapp J., Awawda A., Kouser H., Lui K., Salamon H., Chuyka D., Mahoney W., Furgason J.M. (2025). A Scalable Method for Validated Data Extraction from Electronic Health Records with Large Language Models. medRxiv.

[19] Konet A., Thomas I., Gartlehner G., Kahwati L., Hilscher R., Kugley S., Crotty K., Viswanathan M., Chew R. (2024). Performance of two large language models for data extraction in evidence synthesis. Res. Synth. Methods.

[20] Ntinopoulos V., Rodriguez Cetina Biefer H., Tudorache I., Papadopoulos N., Odavic D., Risteski P., Haeussler A., Dzemali O. (2025). Large language models for data extraction from unstructured and semi-structured electronic health records: A multiple model performance evaluation. BMJ Health Care Inform..

[21] Khan M.A., Ayub U., Naqvi S.A.A., Khakwani K.Z.R., Sipra Z.b.R., Raina A., Zhou S., He H., Saeidi A., Hasan B. (2025). Collaborative large language models for automated data extraction in living systematic reviews. J. Am. Med. Inform. Assoc..

[22] Lai H., Liu J., Bai C., Liu H., Pan B., Luo X., Hou L., Zhao W., Xia D., Tian J. (2025). Language models for data extraction and risk of bias assessment in complementary medicine. npj Digit. Med..

[23] Liu J., Lai H., Zhao W., Huang J., Xia D., Liu H., Luo X., Wang B., Pan B., Hou L. (2025). AI-driven evidence synthesis: Data extraction of randomized controlled trials with large language models. Int. J. Surg..

[24] Courvoisier D.S., Buitrago-Garcia D., Buclin C.P., Burgisser N., Iudici M., Mongin D. (2025). Beyond Human Gold Standards: A Multimodel Framework for Automated Abstract Classification and Information Extraction. Res. Synth. Methods.

[25] van der Loo W., van der Valk V., van den Broek T., Atsma D., Staring M., Scherptong R. (2025). Large Language Models for Structured Cardiovascular Data Extraction: A Foundation for Scalable Research and Clinical Applications. Eur. Heart J. Digit. Health.

[26] Poser P.L., Klimas R., Luerweg J., Reuter E., Hanefeld C., Gold R., Salmen A., Motte J. (2026). Improving Reliability and Accuracy of Structured Data Extraction Using a Consensus Large-Language Model Approach: A Use Case Description in Multiple Sclerosis. Front. Artif. Intell..

[27] Polzak C., Lozano A., Sun M.W., Burgess J., Zhang Y., Wu K., Yeung-Levy S. (2025). Can Large Language Models Match the Conclusions of Systematic Reviews?. arXiv.

[28] Mao G., Snyder W., Chinthala A.S., Singh A., Obeng-Gyasi B., Potts A.J., Jackson L.R., Ortiz Rodriguez K.J., Singh R.S. (2026). Benchmarking Agreement Between Large Language Models and Published Clinical Trial Conclusions Across Four Artificial Intelligence Platforms. Sci. Rep..

[29] Murin M. (2026). Measuring the Sensitivity of LLM-Based Structured Extraction to Prompt, Model, and Schema Choices in Clinical Discharge Summaries. arXiv.

[30] Peng Z., Wu X., Qin Z., Doi S.A., Furuya-Kanamori L., Hong Y., Lin L., Chu H., Xu C., Liu M. (2025). Accuracy of Large Language Models in Data Extraction from Randomized Controlled Trials in Sleep Medicine: A Proof-of-Concept Study. Sleep Med. Rev..

[31] Passweg L.P., Schwenke J.M., Schönenberger C.M., Locher F., Picker J., Dieterle M., Thiele B., Hasler D., Danelli A., Schmitt A.M. (2025). Data Extraction from Oncology Imaging Reports by Large Language Models: A Comparative Accuracy Study. medRxiv.

[32] Jain N., Suh H., Adeyinka S., Roseman L., Allsop A. (2025). Multi-LLM Thematic Analysis with Dual Reliability Metrics: Combining Cohen’s Kappa and Semantic Similarity for Qualitative Research Validation. arXiv.

[33] Haldar R., Hockenmaier J. (2025). Rating Roulette: Self-Inconsistency in LLM-As-A-Judge Frameworks. arXiv.

[34] James J. Counting on Consensus: Selecting the Right Inter-Annotator Agreement Metric for NLP Annotation and Evaluation. Proceedings of the Fifteenth Language Resources and Evaluation Conference (LREC 2026).

[35] Bukhari K., Rodriguez-Monguio R., Lopez-Bermudez B., Yamaki J., Brown L.M., Beuttler R., Ong J.C.L., Seoane-Vazquez E. (2025). When AI Meets the FDA: An Evaluation of Large Language Models Performance in Regulatory and Clinical Trial Data Extraction, Synthesis, and Analysis. medRxiv.

[36] Sun D., Hadjiiski L., Bruno G., Gormley J., Chan H.P., Caoili E.M., Cohan R.H., Alva A., Mihalcea R., Zhou C. (2025). Evaluating the reliability of large language models for clinical data extraction in bladder cancer prognosis. Sci. Rep..

[37] Hsu H.Y., Chen L.W., Hsu W.T., Hsieh Y.W., Chang S.S. (2025). Extracting Clinical Guideline Information Using Two Large Language Models: Evaluation Study. J. Med. Internet Res..

[38] Volkmer S., Glück A., Meyer-Lindenberg A., Schwarz E., Hirjak D. (2025). Validating large language models against manual information extraction from case reports of drug-induced parkinsonism in patients with schizophrenia spectrum and mood disorders: A proof of concept study. Schizophr.

[39] Mahbub M., Dams G.M., Arnold J., Rizy C., Srinivasan S., Fielstein E.M., Aghevli M.A., Craig K.L., Oliva E.M., Erdos J. (2026). A Multi-Stage Validation Framework for Trustworthy Large-scale Clinical Information Extraction using Large Language Models. arXiv.

[40] Borse N.S., Chatta Subramaniam R., Rebello N.S. (2025). Investigation of the Inter-Rater Reliability between Large Language Models and Human Raters in Qualitative Analysis. Physics Education Research Conference Proceedings.

[41] Buch E.R., Santarnecchi E., Antal A., Born J., Celnik P.A., Classen J., Gerloff C., Hallett M., Hummel F.C., Nitsche M.A. (2017). Effects of tDCS on motor learning and memory formation: A consensus and critical position paper. Clin. Neurophysiol..

[42] Pantovic M., Albuquerque L.L.d., Mastrantonio S., Pomerantz A.S., Wilkins E.W., Riley Z.A., Guadagnoli M.A., Poston B. (2023). Transcranial Direct Current Stimulation of Primary Motor Cortex over Multiple Days Improves Motor Learning of a Complex Overhand Throwing Task. Brain Sci..

[43] Fregni F., Boggio P.S., Santos M.C., Lima M., Vieira A.L., Rigonatti S.P., Silva M.T.A., Barbosa E.R., Nitsche M.A., Pascual-Leone A. (2006). Noninvasive cortical stimulation with transcranial direct current stimulation in Parkinson’s disease. Mov. Disord..

[44] Valentino F., Cosentino G., Brighina F., Pozzi N.G., Sandrini G., Fierro B., Savettieri G., D’Amelio M., Pacchetti C. (2014). Transcranial direct current stimulation for treatment of freezing of gait: A cross-over study. Mov. Disord..

[45] Lee S.a., Kim M.K. (2021). The Effect of Transcranial Direct Current Stimulation Combined with Visual Cueing Training on Motor Function, Balance, and Gait Ability of Patients with Parkinson’s Disease. Medicina.

[46] da Silva D.C.L., Lemos T., de Sá Ferreira A., Horsczaruk C.H.R., Pedron C.A., de Carvalho Rodrigues E., de Oliveira L.A.S. (2018). Effects of Acute Transcranial Direct Current Stimulation on Gait Kinematics of Individuals with Parkinson Disease. Top. Geriatr. Rehabil..

[47] Putzolu M., Pelosin E., Ogliastro C., Lagravinese G., Bonassi G., Ravaschio A., Abbruzzese G., Avanzino L. (2018). Anodal tDCS over prefrontal cortex improves dual-task walking in Parkinsonian patients with freezing. Mov. Disord..

[48] Wong P.L., Yang Y.R., Huang S.F., Fuh J.L., Chiang H.L., Wang R.Y. (2022). Transcranial Direct Current Stimulation on Different Targets to Modulate Cortical Activity and Dual-Task Walking in Individuals with Parkinson’s Disease: A Double Blinded Randomized Controlled Trial. Front. Aging Neurosci..

[49] Lima de Albuquerque L., Pantovic M., Clingo M., Fischer K., Jalene S., Landers M., Mari Z., Poston B. (2020). An Acute Application of Cerebellar Transcranial Direct Current Stimulation Does Not Improve Motor Performance in Parkinson’s Disease. Brain Sci..

[50] de Albuquerque L.L., Pantovic M., Clingo M., Fischer K., Jalene S., Landers M., Mari Z., Poston B. (2023). A Single Application of Cerebellar Transcranial Direct Current Stimulation Fails to Enhance Motor Skill Acquisition in Parkinson’s Disease: A Pilot Study. Biomedicines.

[51] Duan Z., Zhang C. (2024). Transcranial direct current stimulation for Parkinson’s disease: Systematic review and meta-analysis of motor and cognitive effects. npj Park. Dis..

[52] Harris D.M., Latella C., Tripodi N., O’Bryan S.J. (2025). Exploring Non-invasive Brain Stimulation Effects on Physical Outcomes in People with Parkinson’s Disease: An Umbrella Evidence Mapping Review with Meta-analyses. Neurorehabil. Neural Repair.

[53] Armstrong M.J., Okun M.S. (2020). Diagnosis and Treatment of Parkinson Disease: A Review. JAMA.

[54] de Oliveira P.C.A., de Araújo T.A.B., de Silva Machado D.G., Rodrigues A.C., Bikson M., Andrade S.M., Okano A.H., Simplicio H., Pegado R., Morya E. (2021). Transcranial Direct Current Stimulation on Parkinson’s Disease: Systematic Review and Meta-Analysis. Front. Neurol..

[55] Wang A., Singh A., Michael J., Hill F., Levy O., Bowman S.R. GLUE: A Multi-Task Benchmark and Analysis Platform for Natural Language Understanding. Proceedings of the BlackboxNLP@EMNLP.

[56] Wang A., Pruksachatkun Y., Nangia N., Singh A., Michael J., Hill F., Levy O., Bowman S.R. (2019). SuperGLUE: A Stickier Benchmark for General-Purpose Language Understanding Systems. arXiv.

[57] Wang Y., Ma X., Zhang G., Ni Y., Chandra A., Guo S., Ren W., Arulraj A., He X., Jiang Z. (2024). MMLU-Pro: A More Robust and Challenging Multi-Task Language Understanding Benchmark. arXiv.

[58] Kweon S., Kim J., Kwak H., Cha D., Yoon H., Kim K., Yang J., Won S., Choi E. EHRNoteQA: An LLM Benchmark for Real-World Clinical Practice Using Discharge Summaries. Proceedings of the Advances in Neural Information Processing Systems 37.

[59] Soldaini L. (2019). The Knowledge and Language Gap in Medical Information Seeking. ACM SIGIR Forum.

[60] Muhamad W., Suhardi, Bandung Y. (2022). Transforming OpenAPI Specification 3.0 documents into RDF-based semantic web services. J. Big Data.

[61] S R.K., Chandrasekar D., T P.K., C T. (2024). Streamlining Access to ClinicalTrials.gov APIs Retrieval of Bulk Data Automation. Int. J. Progress. Res. Eng. Manag. Sci..

[62] Rey A., Freitag M., Neumann T. (2023). Seamless Integration of Parquet Files into Data Processing. Datenbanksysteme fur Business, Technologie und Web.

[63] Nelli F. (2015). The Pandas Library—An Introduction. Python Data Analytics.

[64] van der Walt S., Colbert S.C., Varoquaux G. (2011). The NumPy Array: A Structure for Efficient Numerical Computation. Comput. Sci. Eng..

[65] Khan M.A., Ayub U., Naqvi S.A.A., Khakwani K.Z.R., Sipra Z.b.R., Raina A., Zou S., He H., Hossein S.A., Hasan B. (2024). Collaborative Large Language Models for Automated Data Extraction in Living Systematic Reviews. medRxiv.

[66] Patel D., Timsina P., Raut G., Freeman R., Levin M.A., Nadkarni G.N., Glicksberg B.S., Klang E. (2024). Exploring Temperature Effects on Large Language Models Across Various Clinical Tasks. medRxiv.

[67] Grattafiori A., Dubey A., Jauhri A., Pandey A., Kadian A., Al-Dahle A., Letman A., Mathur A., Schelten A., Vaughan A. (2024). The Llama 3 Herd of Models. arXiv.

[68] Liu J., Shen D., Zhang Y., Dolan B., Carin L., Chen W. (2021). What Makes Good In-Context Examples for GPT-3?. DeeLIO Workshop at ACL.

[69] Ribary M., Krause P., Orban M., Vaccari E., Wood T. (2023). Prompt Engineering and Provision of Context in Domain Specific Use of GPT. Front. Artif. Intell. Appl..

[70] Yousuf R.B., Defelice N., Sharma M., Xu S., Ramakrishnan N. (2024). LLM Augmentations to support Analytical Reasoning over Multiple Documents. Proceedings of the 2024 IEEE International Conference on Big Data (BigData), Washington, DC, USA, 15–18 December 2024.

[71] Mondorf P., Plank B. (2024). Beyond Accuracy: Evaluating the Reasoning Behavior of Large Language Models—A Survey. arXiv.

[72] Chen X., Xiang J., Lu S., Liu Y., He M., Shi D. (2024). Evaluating large language models in medical applications: A survey. arXiv.

[73] Brown T.B., Mann B., Ryder N., Subbiah M., Kaplan J., Dhariwal P., Neelakantan A., Shyam P., Sastry G., Askell A. (2020). Language Models are Few-Shot Learners. Adv. Neural Inf. Process. Syst..

[74] Chen B., Zhang Z., Langrené N., Zhu S. (2025). Unleashing the potential of prompt engineering for large language models. Patterns.

[75] Velasquez-Henao J.D., Franco-Cardona C.J., Cadavid-Higuita L. (2023). Prompt Engineering: A methodology for optimizing interactions with AI-Language Models in the field of engineering. Dyna.

[76] Landis J.R., Koch G.G. (1977). The measurement of observer agreement for categorical data. Biometrics.

[77] Wilkins E.W., Young R.J., Houston D., Kawana E., Lopez Mora E., Sunkara M.S., Riley Z.A., Poston B. (2024). Non-Dominant Hemisphere Excitability Is Unaffected during and after Transcranial Direct Current Stimulation of the Dominant Hemisphere. Brain Sci..

[78] Wilkins E.W., Young R.J., Davidson R., Krider R., Alhwayek G., Park J.A., Parikh A.C., Riley Z.A., Poston B. (2025). The Influence of Transcranial Alternating Current Stimulation on the Excitability of the Unstimulated Contralateral Primary Motor Cortex. Brain Sci..

[79] Feinstein A.R., Cicchetti D.V. (1990). High agreement but low kappa: I. The problems of two paradoxes. J. Clin. Epidemiol..

[80] Yadav T., Tekale A., Chong J., Masum M. (2026). Understanding Tradeoffs in Clinical Text Extraction: Prompting, Retrieval-Augmented Generation, and Supervised Learning on Electronic Health Records. Algorithms.

[81] Borah R., Brown A.W., Capers P.L., Kaiser K.A. (2017). Analysis of the time and workers needed to conduct systematic reviews of medical interventions using data from the PROSPERO registry. BMJ Open.

[82] Wittlinger S., Meerjansen J., Wolf F., Wiest I.C., Ebert M.P., Siegel F., Belle S. (2026). Multi-LLM Disagreement as a Scalable Detector of Human Annotation Errors in Structured Data from Clinical Free-Text. medRxiv.

[83] Zhang B., Liu Z., Cherry C., Firat O. When Scaling Meets LLM Finetuning: The Effect of Data, Model and Finetuning Method. Proceedings of the International Conference on Learning Representations.

[84] Beaulieu-Jones B., Brenner S. Leveraging Foundational Models in Computational Biology: Validation, Understanding, and Innovation. Proceedings of the Pacific Symposium on Biocomputing.

[85] Fleurence R.L., Bian J., Wang X., Xu H., Dawoud D., Higashi M., Chhatwal J. (2024). Generative AI for Health Technology Assessment: Opportunities, Challenges, and Policy Considerations. arXiv.

[86] Kolluri S., Lin J., Liu R., Zhang Y., Zhang W. (2022). Machine Learning and Artificial Intelligence in Pharmaceutical Research and Development: A Review. AAPS J..

[87] Bhatt A. (2021). Artificial intelligence in managing clinical trial design and conduct: Man and machine still on the learning curve?. Perspect. Clin. Res..

[88] Tao C., Fan X., Yang Y. (2024). Harnessing LLMs for API Interactions: A Framework for Classification and Synthetic Data Generation. arXiv.

[89] Heshmatisafa S., Seppänen M. (2023). Exploring API-driven business models: Lessons learned from Amadeus’s digital transformation. Digit. Bus..

[90] Mervaala E., Kousa I. (2025). Out of Context! Managing the Limitations of Context Windows in ChatGPT-4o Text Analyses. J. Data Min. Digit. Humanit..

[91] Georgiev P., Lei V.I., Burnell R., Bai L., Gulati A., Tanzer G., Vincent D., Pan Z., Wang S., Gemini Team (2024). Gemini 1.5: Unlocking multimodal understanding across millions of tokens of context. arXiv.

[92] Shool S., Adimi S., Saboori Amleshi R., Bitaraf E., Golpira R., Tara M. (2025). A systematic review of large language model (LLM) evaluations in clinical medicine. BMC Med. Inform. Decis. Mak..

[93] Sun Z., Zhang R., Doi S.A., Furuya-Kanamori L., Yu T., Lin L., Xu C. (2024). How good are large language models for automated data extraction from randomized trials?. medRxiv.

[94] Wiest I.C., Ferber D., Zhu J., van Treeck M., Meyer S.K., Juglan R., Carrero Z.I., Paech D., Kleesiek J., Ebert M.P. (2024). Privacy-preserving large language models for structured medical information retrieval. npj Digit. Med..

[95] Wiest I.C., Wolf F., Leßmann M.E., van Treeck M., Ferber D., Zhu J., Boehme H., Bressem K.K., Ulrich H., Ebert M.P. (2024). LLM-AIx: An open source pipeline for Information Extraction from unstructured medical text based on privacy preserving Large Language Models. medRxiv.

[96] Wang L., Ma Y., Bi W., Lv H., Li Y. (2024). An Entity Extraction Pipeline for Medical Text Records Using Large Language Models: Analytical Study. J. Med. Internet Res..

[97] Li D., Kadav A., Gao A., Li R., Bourgon R. (2024). Automated Clinical Data Extraction with Knowledge Conditioned LLMs. arXiv.

[98] Bao G., Ma L., Yi X. (2022). Recent advances on cooperative control of heterogeneous multi-agent systems subject to constraints: A survey. Syst. Sci. Control Eng..

[99] Yue L., Xing S., Chen J., Fu T. (2024). ClinicalAgent: Clinical Trial Multi-Agent System with Large Language Model-based Reasoning. Proceedings of the 15th ACM International Conference on Bioinformatics, Computational Biology and Health Informatics, Shenzhen, China, 22–25 November 2024.

[100] Zuo K., Jiang Y., Mo F., Lio P. (2024). KG4Diagnosis: A Hierarchical Multi-Agent LLM Framework with Knowledge Graph Enhancement for Medical Diagnosis. arXiv.

[101] Trirat P., Jeong W., Hwang S.J. (2024). AutoML-Agent: A Multi-Agent LLM Framework for Full-Pipeline AutoML. arXiv.

[102] Schroeder N.L., Jaldi C.D., Zhang S. (2025). Large Language Models with Human-In-The-Loop Validation for Systematic Review Data Extraction. arXiv.

[103] Wang Z., Cao L., Danek B., Jin Q., Lu Z., Sun J. (2025). Accelerating clinical evidence synthesis with large language models. npj Digit. Med..

[104] Kataoka Y., Takayama T., Yoshimura K., So R., Tsujimoto Y., Yamagishi Y., Takagi S., Furukawa Y., Sakata M., Bašić D. (2025). Automating the data extraction process for systematic reviews using GPT-4o and o3. Res. Synth. Methods.

[105] Cao C., Arora R., Cento P., Budak A., Manta K., Farahani E., Cecere M., Selemon A., Sang J., Gong L.X. (2025). Automation of Systematic Reviews with Large Language Models (otto-SR). medRxiv.

[106] Emami M., Shirani M. (2025). Comparing the performance of ChatGPT, DeepSeek, and Gemini in systematic and umbrella review tasks over time. J. Am. Dent. Assoc..

[107] Helms Andersen T., Marcussen T.M., Termannsen A.D., Lawaetz T.W.H., Nørgaard O. (2025). Using Artificial Intelligence Tools as Second Reviewers for Data Extraction in Systematic Reviews. Cochrane Evid. Synth. Methods.

[108] Laignelot F., Martin G.L., Ossman M., Pingeon O., Boubaker A., Picovschi E., Kim J., Tannier X., Cohen J.F., Dechartres A. (2026). Large language models show promising performance for some systematic review tasks but call for cautious implementation: A systematic review. J. Clin. Epidemiol..

[109] Lieberum J.L., Toews M., Metzendorf M.I., Heilmeyer F., Siemens W., Haverkamp C., Böhringer D., Meerpohl J.J., Eisele-Metzger A. (2025). Large language models for conducting systematic reviews: On the rise, but not yet ready for use—A scoping review. J. Clin. Epidemiol..

[110] Nyrhi L., Ponkilainen V., Laaksonen J., Kuikka L., Paljakka L., Karjalainen T., Mattila V.M., Kuitunen I. (2026). Large language models for risk-of-bias assessment in randomised clinical trials—A comparative validation study. eBioMedicine.

[111] Babaeipour R., Charest F., Wright M. (2026). AI-assisted protocol information extraction for improved accuracy and efficiency in clinical trial workflows. J. Biomed. Inform..

[112] Shin E., Bhat A.G., Ramanathan M. (2025). Large Language Models for Clinical Trial Protocol Assessments. Clin. Pharmacol. Ther..

[113] Tang T., Li A., Tan X., Ji Q., Si L., Bao L. (2025). Bridging Data Gaps in Oncology: Large Language Models and Collaborative Filtering for Cancer Treatment Recommendations. medRxiv.

[114] Achterberg J., van Dijk B., Meng J., Islam S.U., Epiphaniou G., Maple C., Ding X., Arvanitis T.N., Brouwer S., Haas M. (2026). OpenExtract: Automated Data Extraction for Systematic Reviews in Health. Studies in Health Technology and Informatics.

[115] Chen L., He R., Lu P., Jin Y., Zhou L., Li N., Wu P., Hu B. (2026). Operationalizing Large Language Models for Clinical Research Data Extraction: Methods, Quality Control, and Governance. J. Med. Syst..

